# Supplementary material for: Antagonistic nanobodies implicate mechanism of GSDMD pore formation and potential therapeutic application
Source: Nat Commun. 2024 Sep 26;15:8266. doi: 10.1038/s41467-024-52110-1 (PMC11427689; doi:10.1038/s41467-024-52110-1)

Supplementary Materials for

Antagonistic nanobodies implicate mechanism of GSDMD pore formation and potential therapeutic application

Lisa D.J. Schiffelers[1], Yonas M. Tesfamariam[1], Lea-Marie Jenster[1], Stefan Diehl[1], Sophie C. Binder[1], Sabine Normann[1], Jonathan Mayr[1], Steffen Pritzl[1], Elena Hagelauer[1], Anja Kopp[2,3], Assaf Alon[4], Matthias Geyer[2], Hidde L. Ploegh[4], Florian I. Schmidt[1,4,5*]

[1]Institute of Innate Immunity, Medical Faculty, University of Bonn, 53127 Bonn, Germany
[2]Institute of Structural Biology, Medical Faculty, University of Bonn, 53127 Bonn, Germany
[3]Inflammation Division, The Walter and Eliza Hall Institute of Medical Research, 12 Parkville, VIC 3052, Australia
[4]Whitehead Institute for Biomedical Research, Cambridge, MA 02142, USA
[4]Core Facility Nanobodies, Medical Faculty, University of Bonn, 53127 Bonn, Germany

*Correspondence to: Florian Schmidt, Institute of Innate Immunity, University of Bonn, Venusberg-Campus 1, 53127 Bonn, Germany, E-mail: fschmidt@uni-bonn.de, ORCID: 0000-0002-9979-9769

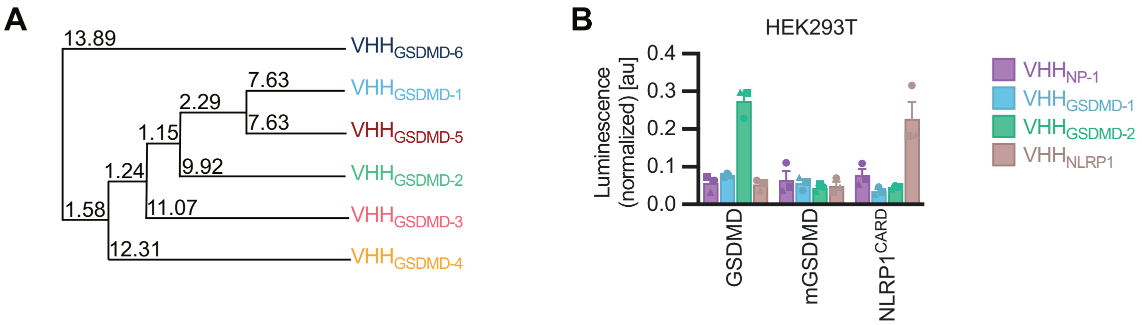

Supplementary Fig. 1. Identification of GSDMD-specific nanobodies. **A** Average distance tree calculated based on the percentage identity between the selected nanobody sequences shown in Fig. 1B. **B** LUMIER assay: HEK293T cells were co-transfected with expression vectors for the specified HA-tagged nanobodies and the indicated protein-Renilla luciferase fusions. 24 h post transfection, cells were lysed and VHH-HA was immunoprecipitated with immobilized anti-HA. Coelenterazine-h was added and luminescence of co-purified Renilla luciferase was measured and normalized to luminescence of lysates. Data represent average values (with individual data points) from three independent experiments ± SEM.

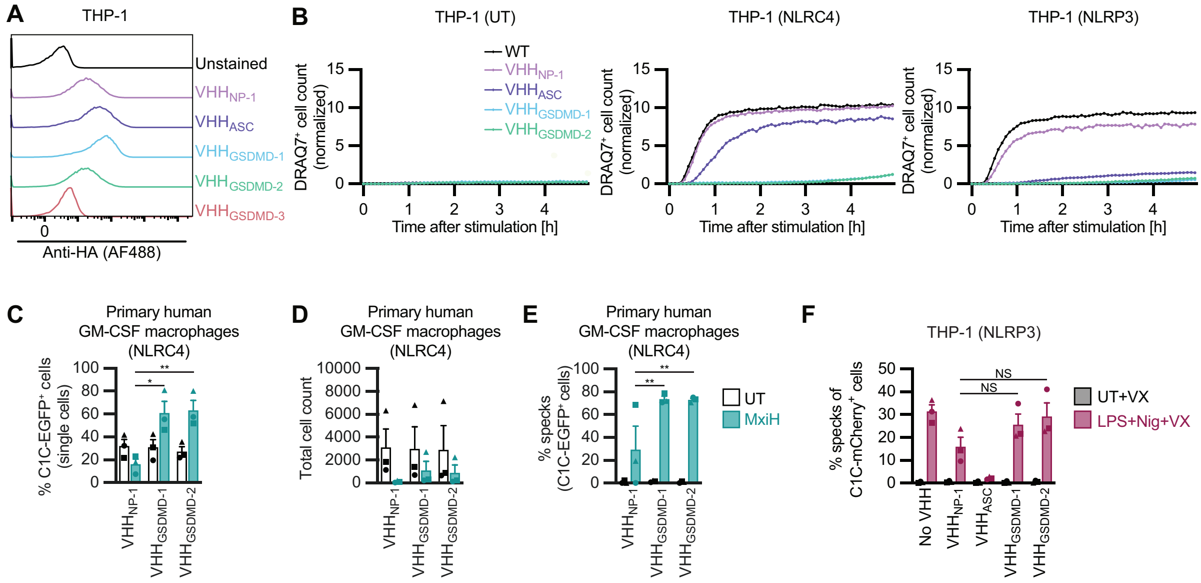

Supplementary Fig. 2. VHH$_{GSDMD-1}$ and VHH$_{GSDMD-2}$ abrogate pyroptosis. A THP-1 cell lines constitutively expressing the indicated HA-tagged nanobodies were fixed, stained for HA, and histograms of the HA signals of a representative experiment are displayed. **B** PMA-differentiated THP-1 macrophages were stimulated with NLRC4 and NLRP3 activators as described in Fig. 2C and 2D, but in the presence of 100 nM DRAQ7. DRAQ7 uptake was monitored over 5 h in an Incucyte Live-Cell Imaging system. Representative images (of n=3) after 1 h from the same experiment are displayed in Fig. 2G. **C-E** Primary GM-CSF-differentiated monocyte-derived human macrophages were transduced and stimulated as described in Fig. 2H-K. 1 h post treatment, cells were harvested, fixed, and analyzed by flow cytometry to determine the fraction of C1C-EGFP$^+$ and thus VHH-expressing cells (C), cell count over 30 s (D), and the fraction of C1C-EGFP$^+$ cells assembling ASC specks (E). **F** THP-1 cell lines expressing C1C-mCherry (dox-inducible) as well as the indicated VHH-EGFP fusions (constitutively) were differentiated with PMA, treated with dox for 24 h, and subjected to NLRP3 stimulation with LPS and nigericin (Nig) as described in Fig. 2D, in presence of 40 µM VX. Cells were harvested and ASC specks were quantified by flow cytometry. Data represent average values (with individual data points) from three independent donors ± SEM. NS, not significant; $*P < 0.05$, $**P < 0.01$ (unpaired two-tailed Student's *t*-test).

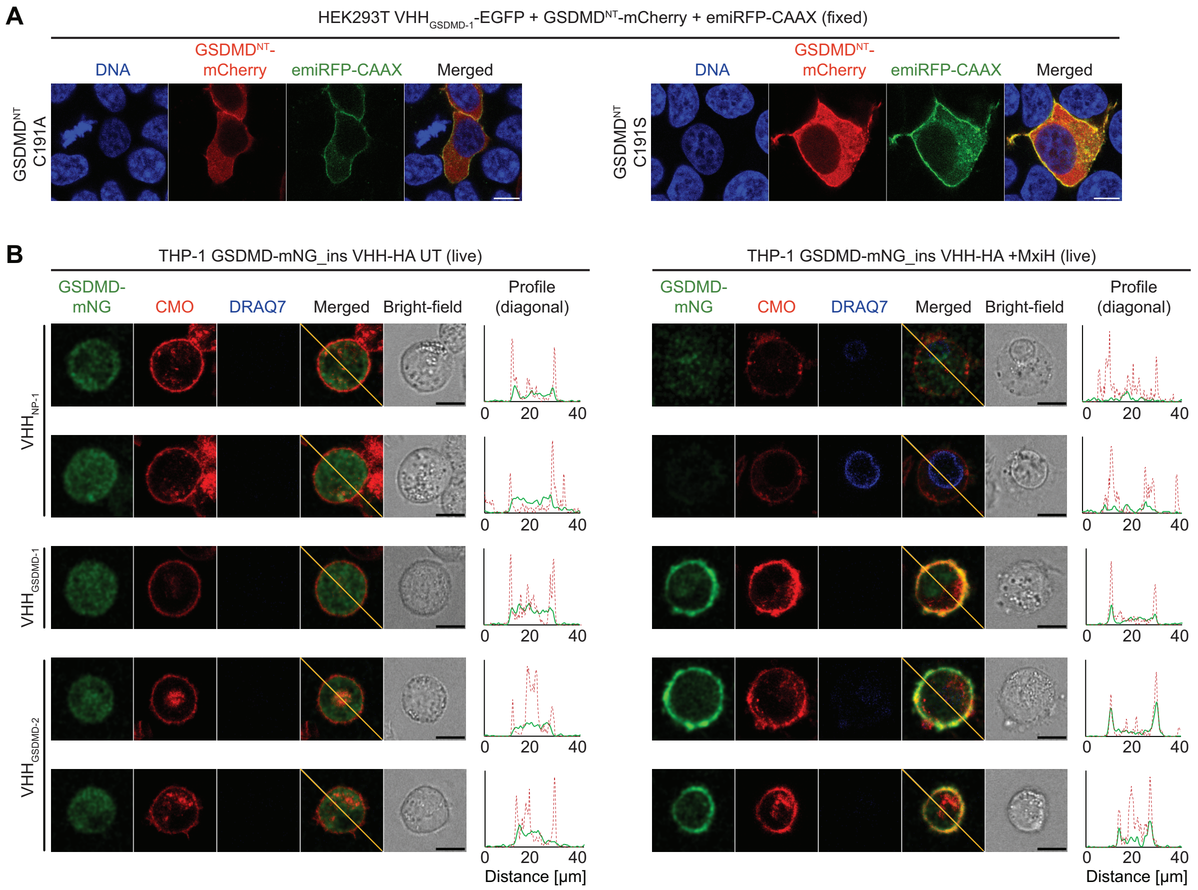

Supplementary Fig. 3. Nanobodies preventing oligomerization still allow membrane localization of GSDMD^{NT}. A HEK293T cells stably expressing VHH$_{GSDMD-1}$-EGFP were transfected with expression vectors for the plasma membrane marker emiRFP670-CAAX as well as the indicated GSDMD variants fused to mCherry. Additional representative images of the experiment in Figure 3B are displayed. **B** PMA-differentiated THP-1 cells expressing GSDMD-mNG_ins and the indicated HA-tagged nanobodies were stained with CMO, left untreated (left) or stimulated with MxiH (right), and recorded as described for Fig. 4C. Additional representative images and intensity profiles of the experiment in Fig. 4C are displayed. Images representative of at least three independent experiments are displayed. Scale bars, 10 µm.

A

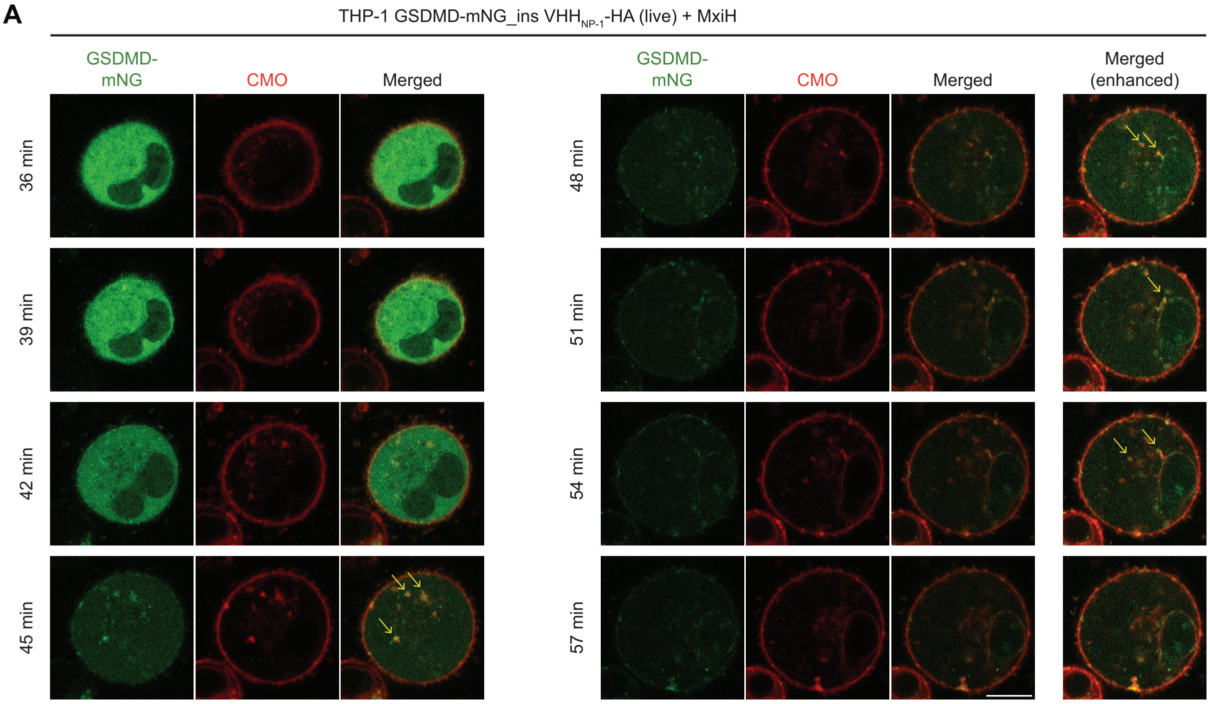

B

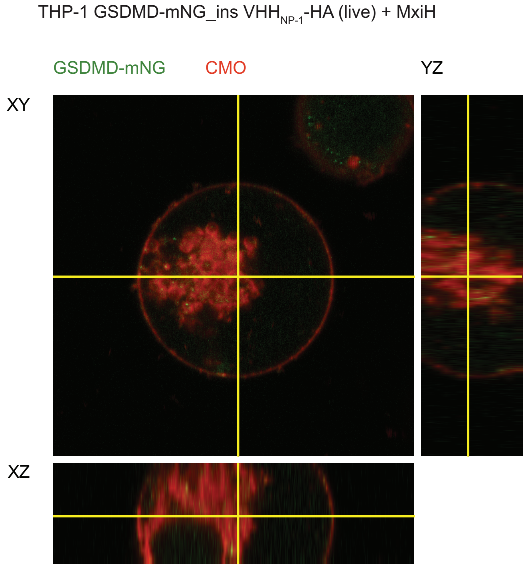

Supplementary Fig. 4. GSDMD^{NT}-mNG localizes to internal structures after pore formation.

PMA-differentiated THP-1 cells expressing GSDMD-mNG_ins and VHH$_{NP-1}$-HA were stained with CMO and stimulated with MxiH as in Fig. S3B. **A** Stimulated cells were followed over time by live cell confocal microscopy (3 min intervals; time post treatment indicated). Merged images with enhanced brightness are displayed for the later time point on the right. Intracellular vesicular structures positive for GSDMD-mNG and CMO are highlighted with yellow arrows. All time points of the same cell are displayed in Movie S1; a second cell from the same experiment is displayed in Movie S2. Images representative of at least three independent experiments are displayed. Scale bars, 10 µm. **B** A Z-stack containing a representative cell was recorded 60 min post stimulation. An XY section as well as matching XZ and YZ sections are shown for the cell in the middle.

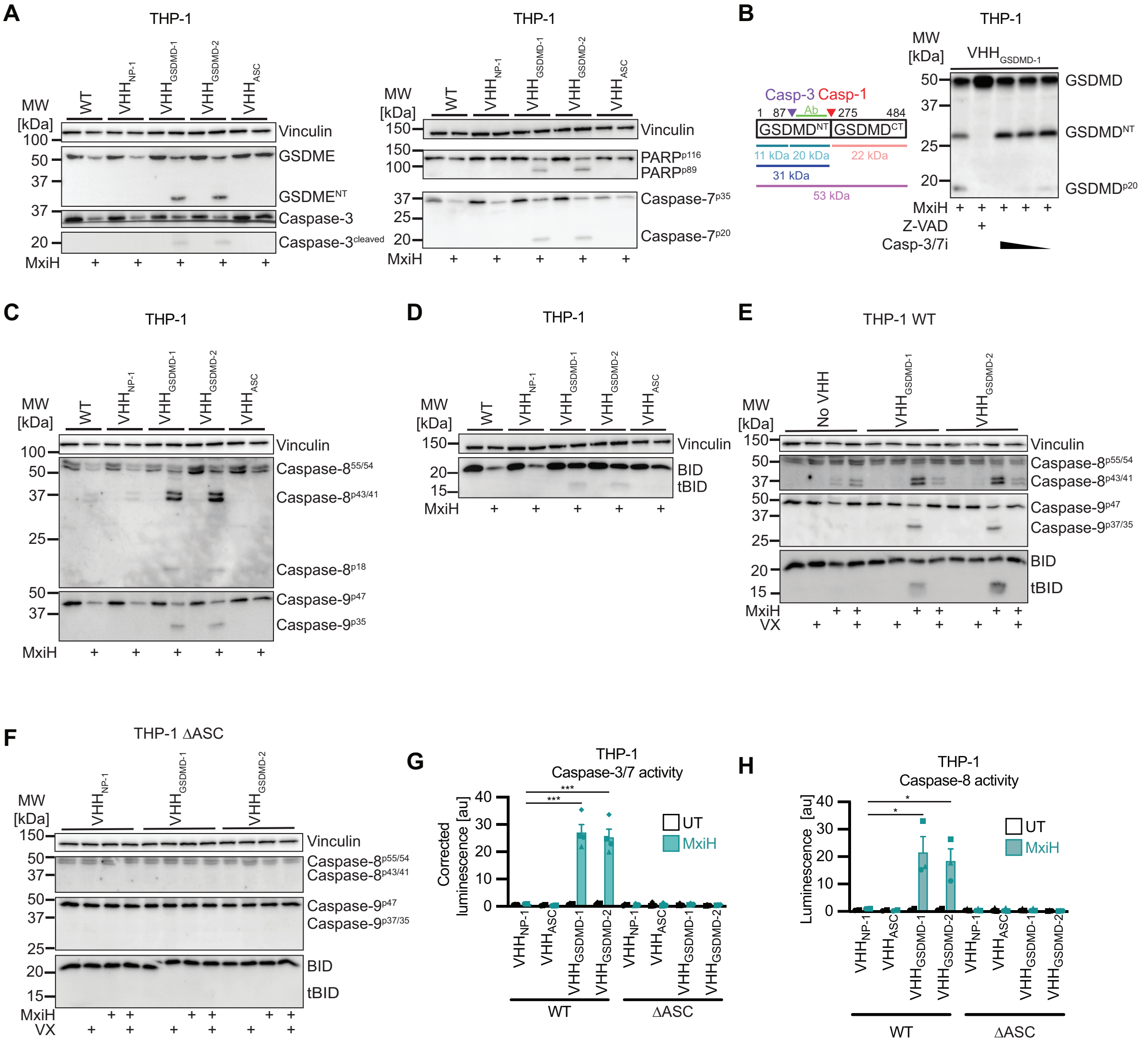

Supplementary Fig. 5. Inhibition of pore formation by antagonistic GSDMD nanobodies triggers caspase-1-dependent apoptosis. PMA-differentiated THP-1 WT (A-E, G, H) or THP-1 ΔASC (F-H) cells constitutively expressing the indicated HA-tagged nanobodies were stimulated with MxiH for 1 h as described in Fig. 2C. Experiments were performed in the presence of 40 µM VX, 50 µM Z-VAD, or caspase-3/7 inhibitor (30, 20, and 4 µM) as indicated. **A-F** Proteins from cell lysates were separated by SDS-PAGE and analyzed by immunoblot with the indicated antibodies. Immunoblots with caspase-3 and cleaved caspase-3 antibodies were prepared from the same (cut) membrane and developed separately, with a longer exposure for caspase-3^{cleaved} (A). Caspase-3 (Casp-3) and caspase-1 (Casp-1) cleavage sites in GSDMD, resulting fragments, and the peptide used to raise the GSDMD antibody (Ab) are indicated next to the GSDMD immunoblots (B). **G-H** Cells and supernatants were harvested to measure caspase-3/7 (G) or caspase-8 (H) activity using Caspase-Glo assays. Luminescence was corrected for total cell numbers per sample using CTB values (G, H). Representative immunoblots of at least three independent experiments are displayed. Caspase activity data represents average values (with individual data points) from three independent experiments ± SEM. $*P < 0.05$, and $***P < 0.001$ (unpaired two-tailed Student's *t*-test).

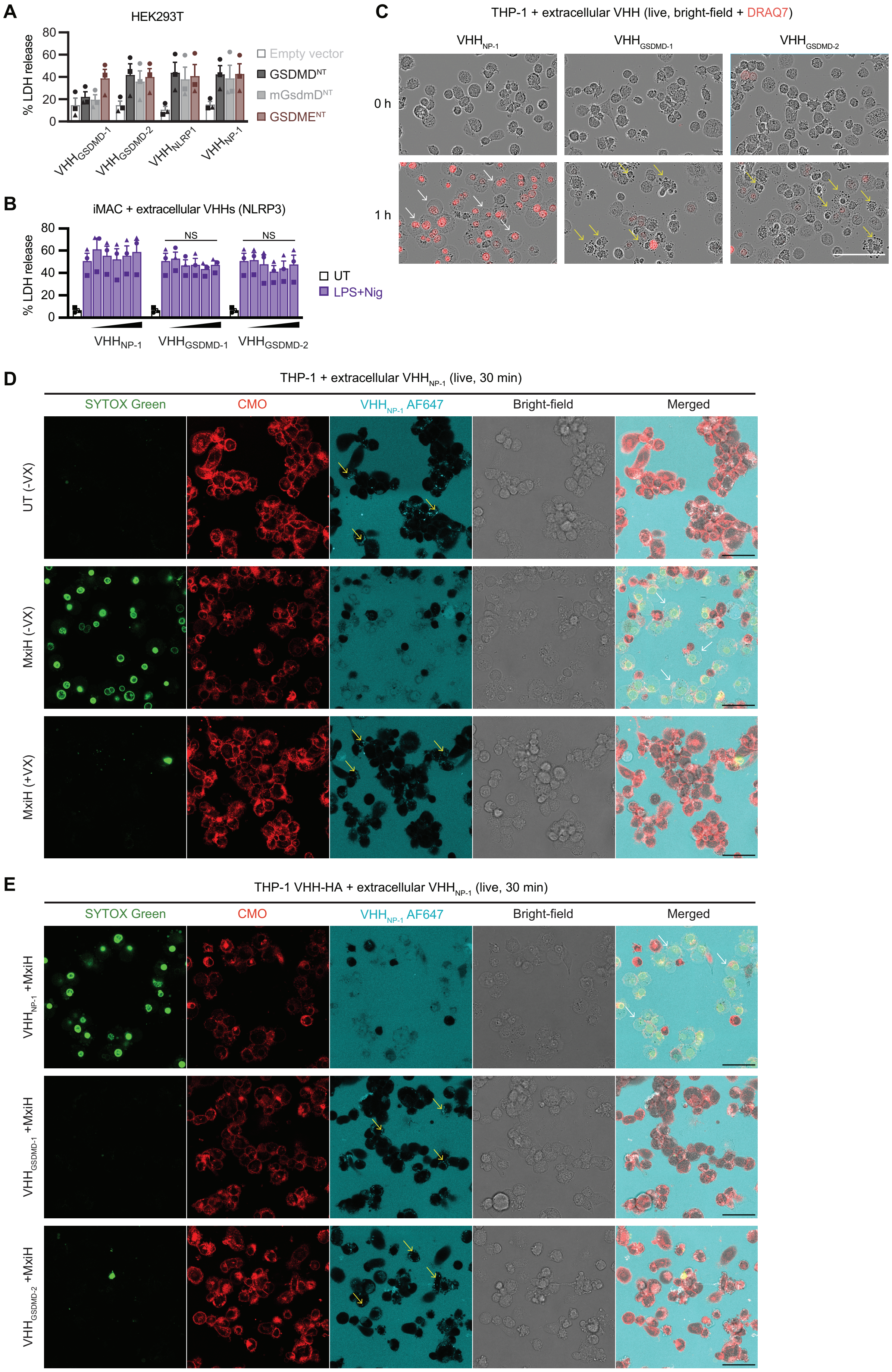
A
HEK293T
% LDH release
80
60
40
20
0
Empty vector
GSDMDNT
mGsdmDNT
GSDMENT
VHHGSDMD-1
VHHGSDMD-2
VHHNLRP1
VHHNP-1
B
iMAC + extracellular VHHs (NLRP3)
% LDH release
NS
NS
UT
LPS+Nig
VHHNP-1
VHHGSDMD-1
VHHGSDMD-2
C
THP-1 + extracellular VHH (live, bright-field + DRAQ7)
VHHNP-1
VHHGSDMD-1
VHHGSDMD-2
0 h
1 h
D
THP-1 + extracellular VHHNP-1 (live, 30 min)
SYTOX Green
CMO
VHHNP-1 AF647
Bright-field
Merged
UT (-VX)
MxiH (-VX)
MxiH (+VX)
E
THP-1 VHH-HA + extracellular VHHNP-1 (live, 30 min)
SYTOX Green
CMO
VHHNP-1 AF647
Bright-field
Merged
VHHNP-1 +MxiH
VHHGSDMD-1 +MxiH
VHHGSDMD-2 +MxiH

Supplementary Fig. 6. Partial inhibition of murine GsdmD^{NT} pore formation and uptake of extracellularly administered recombinant antagonistic GSDMD nanobodies. **A** HEK293T cells were co-transfected with expression vectors for the indicated HA-tagged nanobodies as well as empty vector, GSDMD^{NT}, murine GsdmD^{NT} (mGsdmD^{NT}), or GSDME^{NT}. LDH release was measured 24 h post transfection and normalized to cells lysed in 1% Triton X-100. **B** Murine iMACs were treated with LPS and Nig as described in Fig. 2D in the presence of increasing concentrations of the indicated recombinant nanobodies as in Fig. 5C. LDH release was measured and normalized as in (A). Data represent average values (with individual data points) from three independent experiments ± SEM. **C** PMA-differentiated THP-1 cells were treated with MxiH as described in Fig. 2C, but in the presence of DRAQ7. Cells were recorded with an Incucyte Live-Cell Imaging system and representative images of three independent repeats are displayed. White arrows indicate pyroptotic cells, yellow arrows indicate apoptotic cells. Scale bar, 100 µm. **D, E** THP-1 cells (D) or THP-1 cell lines expressing the indicated HA-tagged VHHs (E) were differentiated with PMA, labeled with CMO, and stimulated with MxiH in the presence of fluorescent VHH_{NP-1} as described in Fig 5I. Cells were recorded by live cell confocal microscopy including bright field recordings and images representative of three independent experiments are displayed. VHH_{NP-1} AF647 in endosomes is indicated with yellow arrows, while cytosolic VHH_{NP-1} AF647 is highlighted with white arrows. Quantification of VHH uptake is displayed in Fig. 5, I and J. Scale bars, 50 µm. NS, not significant (unpaired two-tailed Student's *t*-test).

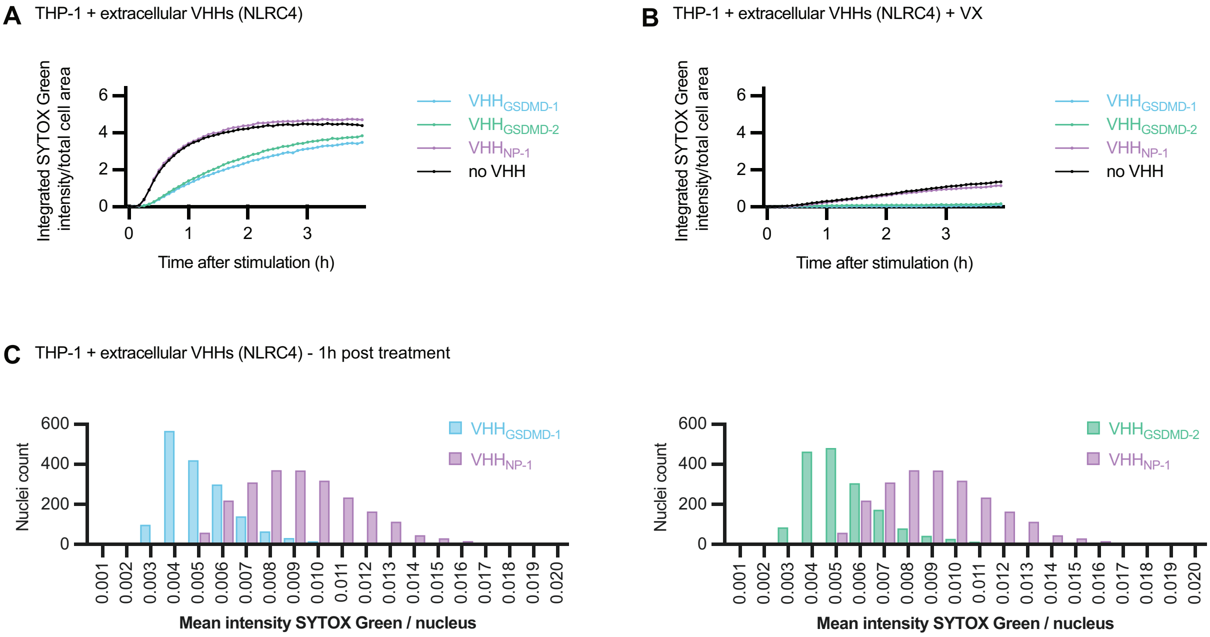

Supplementary Fig. 7. Pyroptoptic cells and cells with transient GSDMD pores take up different amounts of DNA dyes. A-C PMA-differentiated THP-1 cells were treated with MxiH as described in Fig. 2C, but in the presence of SYTOX Green and 200 µg/mL of the indicated nanobodies in the absence (A, C) or presence (B) of VX. Cells were recorded with an Incucyte Live-Cell Imaging system and the integrated SYTOX Green intensity per cell area displayed over time (A, B). SYTOX Green-positive nuclei were identified and the mean fluorescence intensity was extracted. Nuclei were categorized in the indicated bins of SYTOX Green intensity and the resulting histograms displayed (C) to compare dye influx in presence of inhibitory VHH$_{GSDMD-1}$ (left) and VHH$_{GSDMD-1}$ (right) to control nanobody VHH$_{NP-1}$ (pyroptotic cells). Data representative of at least three independent experiments is displayed.

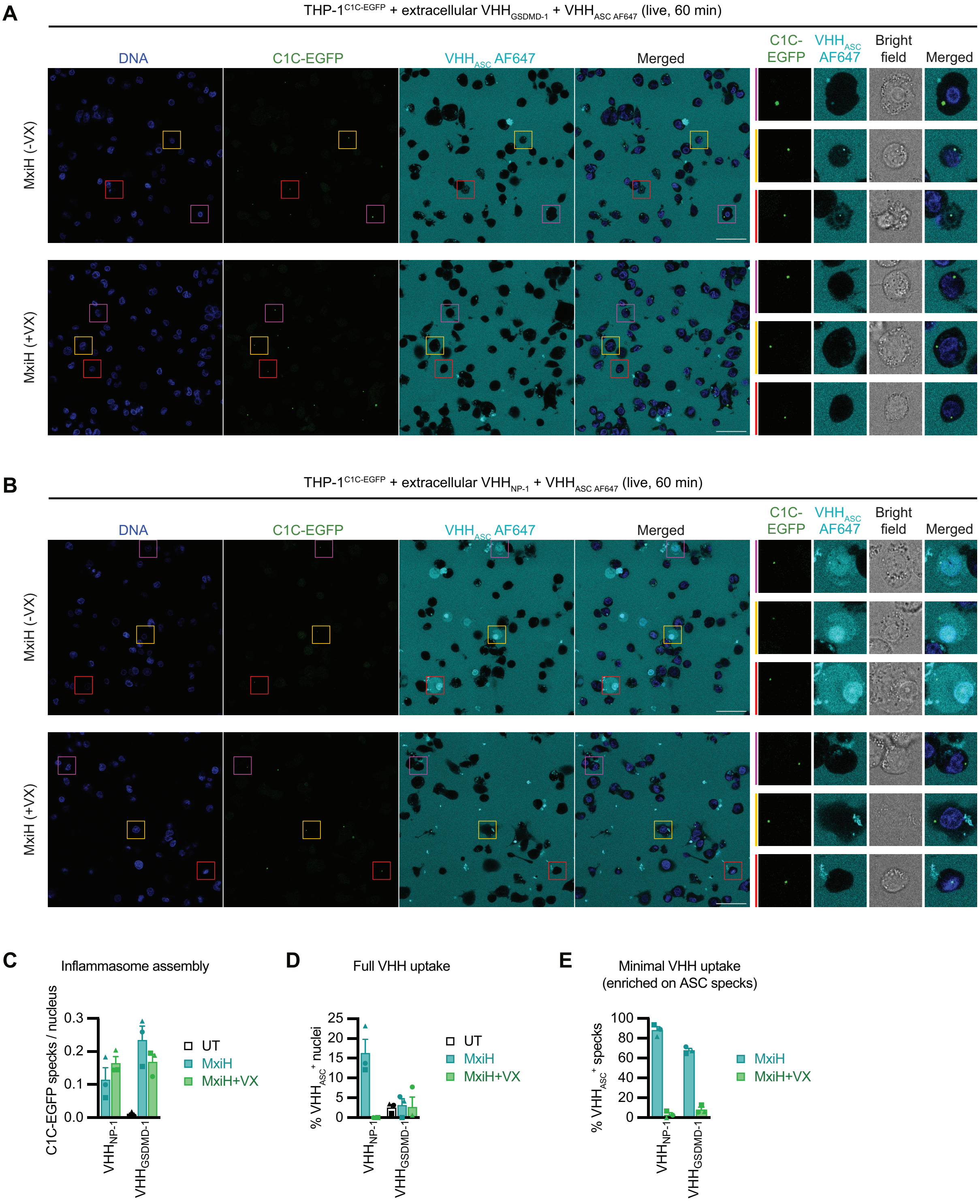
A
THP-1C1C-EGFP + extracellular VHHGSDMD-1 + VHHASC AF647 (live, 60 min)
DNA
C1C-EGFP
VHHASC AF647
Merged
C1C-EGFP
VHHASC AF647
Bright field
Merged
MxiH (-VX)
MxiH (+VX)
B
THP-1C1C-EGFP + extracellular VHHNP-1 + VHHASC AF647 (live, 60 min)
DNA
C1C-EGFP
VHHASC AF647
Merged
C1C-EGFP
VHHASC AF647
Bright field
Merged
MxiH (-VX)
MxiH (+VX)
C
Inflammasome assembly
C1C-EGFP specks / nucleus
0.3
0.2
0.1
0.0
VHHNP-1
VHHGSDMD-1
UT
MxiH
MxiH+VX
D
Full VHH uptake
% VHHASC+ nuclei
25
20
15
10
5
0
VHHNP-1
VHHGSDMD-1
UT
MxiH
MxiH+VX
E
Minimal VHH uptake
(enriched on ASC specks)
% VHHASC+ specks
100
80
60
40
20
0
VHHNP-1
VHHGSDMD-1
MxiH
MxiH+VX

Supplementary Fig. 8. Nanobodies are taken up by pyroptotic cells and cells with transient GSDMD pores. A-E PMA-differentiated THP-1 constitutively expressing C1C-EGFP (THP-1$^{C1C\text{-}EGFP}$) were treated with MxiH as described in Fig. 2C in the presence of 30 µg/mL VHH$_{ASC}$ AF647 as well as 200 µg/mL of VHH$_{GSDMD\text{-}1}$ (A) or VHH$_{NP\text{-}1}$ (B); cells were stimulated in the absence (A-E) or presence of VX (C-E, where indicated). Cells were recorded by live cell confocal microscopy and representative images are displayed. Scale bars, 50 µm. Three exemplary cells with assembled inflammasomes (C1C-EGFP specks) are displayed in color-coded insets to the right. Nuclei and C1C-EGFP specks were detected and the fraction of cells with C1C-EGFP specks (C), the fraction of cells with full VHH$_{ASC}$ AF647 uptake throughout the cell (represented by AF647-positive nuclei) (D), as well as the fraction of C1C-EGFP specks positive for AF647 (indicative of minimal VHH uptake) (E) were quantified and average values from three independent experiments with at least n=250 cells per condition (typically more than 500 cells) are displayed ± SEM.

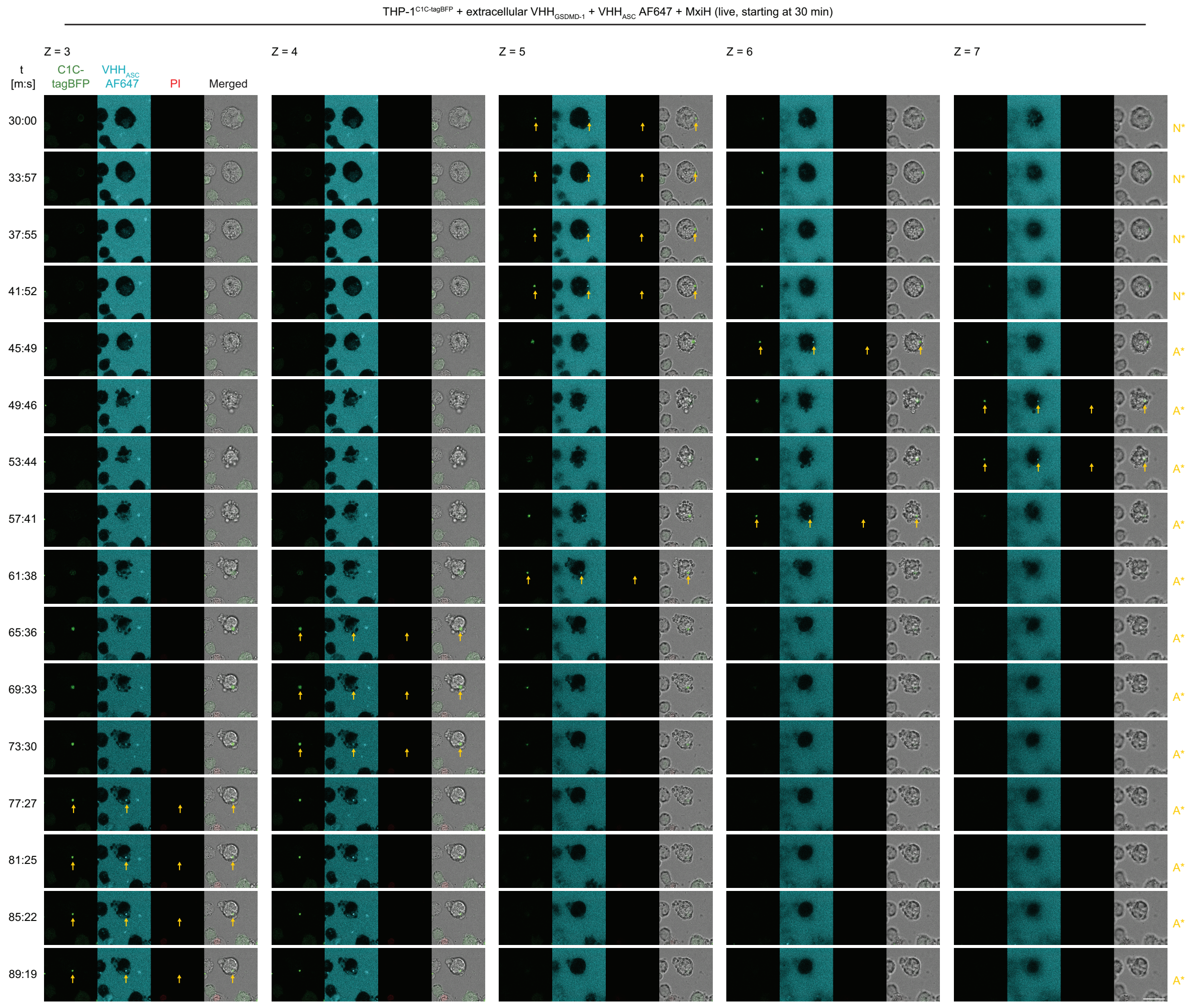

Supplementary Fig. 9. Uptake of nanobodies through transient GSDMD pores precedes apoptosis. PMA-differentiated THP-1$^{C1C\text{-}tagBFP}$ were treated with MxiH in the presence of inhibitory VHH$_{GSDMD\text{-}1}$ and low concentrations of VHH$_{ASC}$ AF647 as in Figure S8, but in the presence of PI. Z stacks of cells with C1C-tagBFP specks were recorded over time by live cell confocal microscopy (starting 30 min post treatment, Z stack planes with a distance of 2 µm). Five Z planes of a representative cell at the indicate time points are displayed. The yellow arrow indicates a C1C-tagBFP speck that moves to the different planes over time. A movie displaying the planes marked with the arrow is supplied as Movie S3. Scale bar, 25 µm. The letters to the right indicated the morphology of the cell, with N=normal, and A=apoptotic. Asterisks indicate that VHH$_{ASC}$ AF647 co-localizes with the C1C-tagBFP speck (indicative of minimal nanobody uptake that precedes apoptosis). The cells remained PI negative (minute uptake through transient GSDMD pores is not detected in confocal slices in the applied experimental conditions); compare to Figure S11 for PI-positive pyroptotic cells.

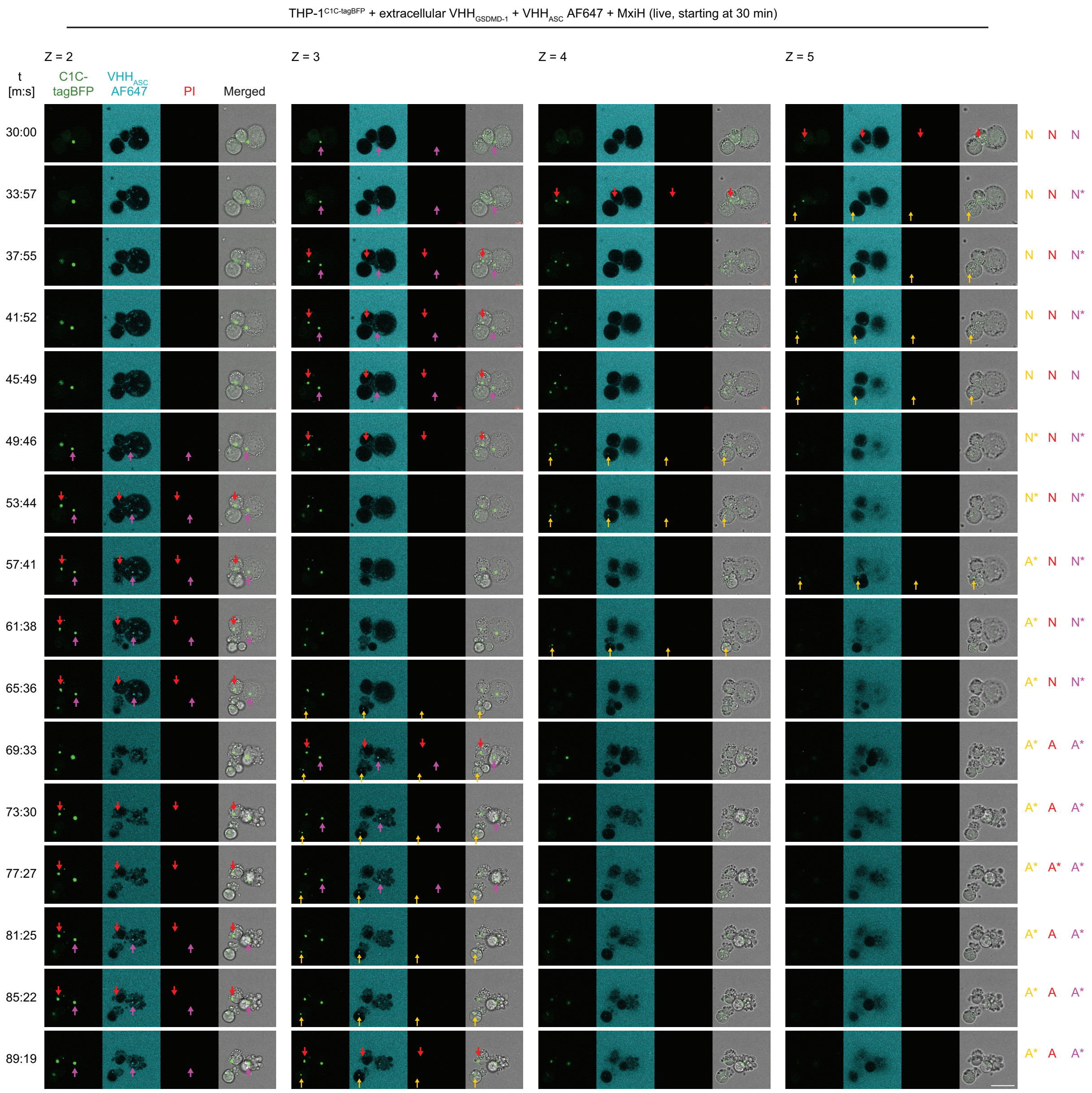

Supplementary Fig. 10. Uptake of nanobodies through transient GSDMD pores precedes apoptosis. PMA-differentiated THP-1$^{C1C\text{-}tagBFP}$ were treated with MxiH in the presence of inhibitory VHH$_{GSDMD\text{-}1}$, low concentrations of VHH$_{ASC}$ AF647, and PI as in Figure S8. Z stacks were recorded over time and four Z planes of representative cells at the indicate time points are displayed. The magenta, yellow, and red arrow indicate individual C1C-tagBFP specks that move to the different planes over time. Movies displaying the planes marked with the magenta or yellow arrow are supplied as Movie S4 and Movie S5, respectively. Scale bar, 25 µm. The letters to the right indicated the morphology of the cell, with N=normal, and A=apoptotic. Asterisks indicate that VHH$_{ASC}$ AF647 co-localizes with the C1C-tagBFP speck (indicative of minimal nanobody uptake that precedes apoptosis). The cells remained PI negative (minute uptake through transient GSDMD pores is not detected in confocal slices in the applied experimental conditions); compare to Figure S11 for PI-positive pyroptotic cells.

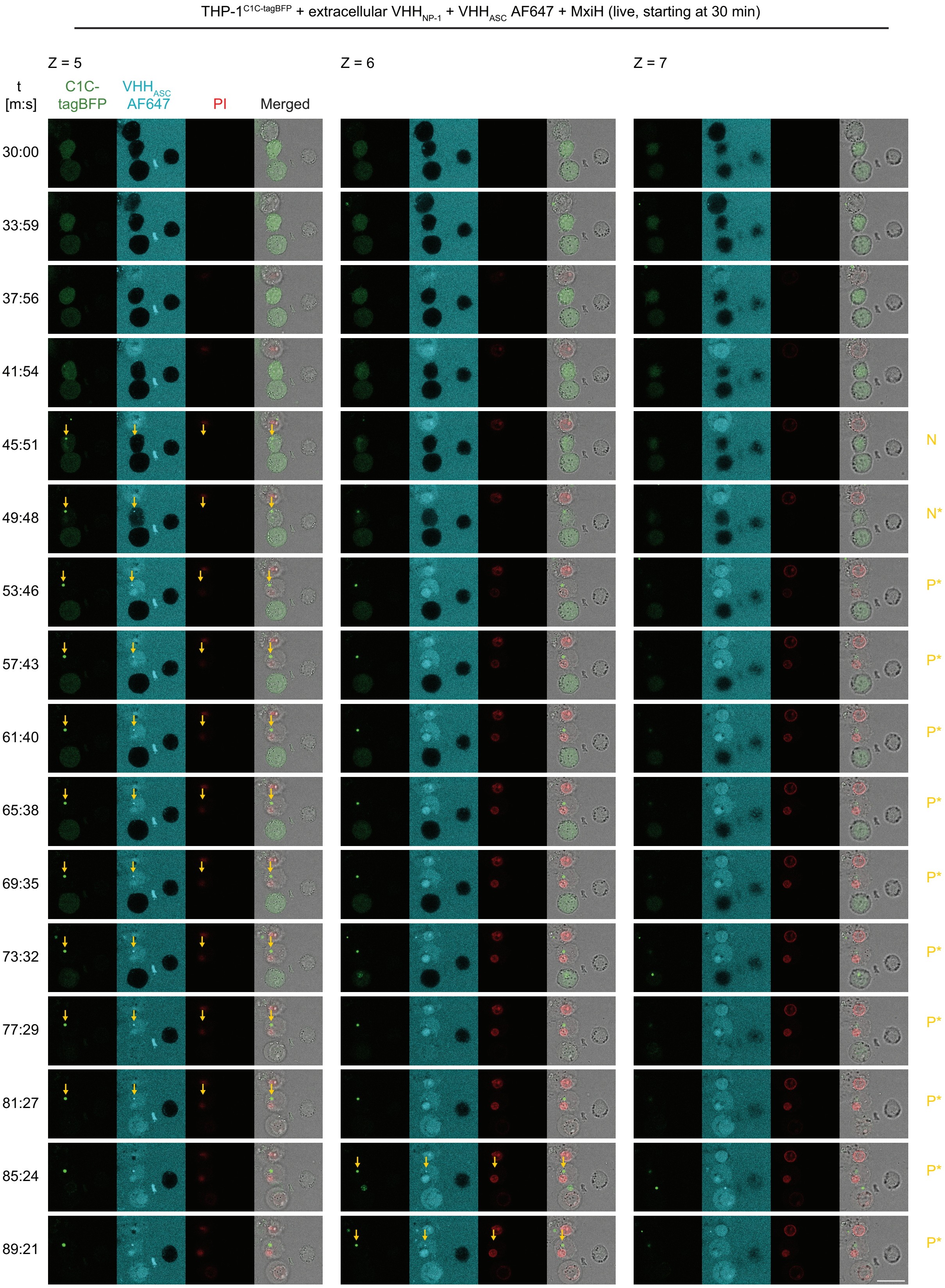

THP-1$^{C1C\text{-}tagBFP}$ + extracellular VHH$_{NP\text{-}1}$ + VHH$_{ASC}$ AF647 + MxiH (live, starting at 30 min)
Z = 5
Z = 6
Z = 7
t [m:s]
C1C-tagBFP
VHH$_{ASC}$ AF647
PI
Merged
30:00
33:59
37:56
41:54
45:51
49:48
53:46
57:43
61:40
65:38
69:35
73:32
77:29
81:27
85:24
89:21
N
N*
P*
P*
P*
P*
P*
P*
P*
P*
P*
P*

Supplementary Fig. 11. Uptake of nanobodies through GSDMD during pyroptosis. PMA-differentiated THP-$1^{C1C\text{-}tagBFP}$ were treated with MxiH in the presence of control $VHH_{NP\text{-}1}$ and low concentrations of VHH_{ASC} AF647 as in Figure S8, but in the presence of PI. Z stacks were recorded over time and three Z planes of representative cells at the indicate time points are displayed. The yellow arrow indicates a C1C-tagBFP speck that moves to the different planes over time. A movie displaying the planes marked with the yellow arrow is supplied as Movie S6. Scale bar, 25 μm. The letters to the right indicated the morphology of the cell, with N=normal, and P=pyroptotic. Asterisks indicate that VHH_{ASC} AF647 co-localizes with the C1C-tagBFP speck. The cells become PI positive concurrent with the strong uptake of VHH_{ASC} AF647.

Supplementary Table 1. Cell lines

Cell line name	Internal cell line #	Lentiviral vector name	Plasmid #
HEK293T WT	H0		
HEK293T VHH$_{GSDMD-1}$-EGFP	H80	pRRL pEF1alpha NA VHH$_{GSDMD-1}$-EGFP Puro	2826
HEK293T VHH$_{GSDMD-2}$-EGFP	H77	pRRL pEF1alpha NA VHH$_{GSDMD-2}$-EGFP Puro	2641
HEK293T VHH$_{NP-1}$-EGFP	H82	pRRL pEF1alpha NA VHH$_{NP-1}$-EGFP Puro	2830
THP-1 WT	T0		
THP-1 VHH$_{GSDMD-1}$-HA	T242	pRRL pEF1alpha NA VHH$_{GSDMD-1}$-HA Puro	2503
THP-1 VHH$_{GSDMD-2}$-HA	T248	pRRL pEF1alpha NA VHH$_{GSDMD-2}$-HA Puro	2818
THP-1 VHH$_{GSDMD-3}$-HA	T250	pRRL pEF1alpha NA VHH $_{GSDMD-3}$-HA Puro	2820
THP-1 VHH$_{NP-1}$-HA	T252	pRRL pEF1alpha NA VHH$_{NP-1}$-HA Puro	2822
THP-1 VHH$_{ASC}$-HA	T253	pRRL pEF1alpha NA VHH$_{ASC}$-HA Puro	2824
THP-1 C1C-mCherry(i)	T60	pInducer20-NA caspase-1 CARD-mCherry	818
THP-1 C1C-mCherry(i) VHH$_{GSDMD-1}$-EGFP	T438	pInducer20-NA caspase-1 CARD-mCherry	818
		pRRL pEF1alpha NA VHH$_{GSDMD-1}$-EGFP Puro	2826
THP-1 C1C-mCherry(i) VHH$_{NP-1}$-EGFP	T440	pInducer20-NA caspase-1 CARD-mCherry	818
		pRRL pEF1alpha NA VHH$_{NP-1}$-EGFP Puro	2830
THP-1 C1C-mCherry(i) VHH$_{ASC}$-EGFP	T441	pInducer20-NA caspase-1 CARD-mCherry	818
		pRRL pEF1alpha NA VHH$_{ASC}$-EGFP Puro	2639
THP-1 C1C-mCherry(i) VHH$_{GSDMD-2}$-EGFP	T442	pInducer20-NA caspase-1 CARD-mCherry	818
		pRRL pEF1alpha NA VHH$_{GSDMD-2}$-EGFP Puro	2641
THP1 C1C-EGFP	T76	pRRL pUbC C1C-EGFP Hyg	846
THP-1 C1C-tagBFP	T276	pRRL pUbC C1C-tagBFP Puro	2993
THP-1 ΔASC 5a1	T137	pLenti CRISPR v2 sgASC 5	765
THP-1 ΔASC VHH$_{GSDMD-1}$-EGFP	T651	pLenti CRISPR v2 sgASC 5	765
		pRRL pEF1alpha NA VHH$_{GSDMD-1}$-EGFP Puro	2826
THP-1 ΔASC VHH$_{NP-1}$-EGFP	T652	pLenti CRISPR v2 sgASC 5	765
		pRRL pEF1alpha NA VHH$_{NP-1}$-EGFP Puro	2830
THP-1 ΔASC VHH$_{ASC}$-EGFP	T653	pLenti CRISPR v2 sgASC 5	765
		pRRL pEF1alpha NA VHH$_{ASC}$-EGFP Puro	2639
THP-1 ΔASC VHH$_{GSDMD-2}$-EGFP	T654	pLenti CRISPR v2 sgASC 5	765
		pRRL pEF1alpha NA VHH$_{GSDMD-2}$-EGFP Puro	2641
THP-1 GSDMD-mNeonGreen_ins(i)	T218	pInducer20 GSDMD-mNeonGreen	2146
THP-1 GSDMD-mNeonGreen_ins(i) VHH$_{GSDMD-1}$-HA	T712	pInducer20 GSDMD-mNeonGreen	2146
		pRRL pEF1alpha NA VHH$_{GSDMD-1}$-HA Puro	2503
THP-1 GSDMD-mNeonGreen_ins(i) VHH$_{GSDMD-2}$-HA	T713	pInducer20 GSDMD-mNeonGreen	2146
		pRRL pEF1alpha NA VHH$_{GSDMD-2}$-HA Puro	2818
THP-1 GSDMD-mNeonGreen_ins(i) VHH$_{NP-1}$-HA	T714	pInducer20 GSDMD-mNeonGreen	2146

Uncropped immunoblots from supplementary figures:

Figure S4A and S5C*

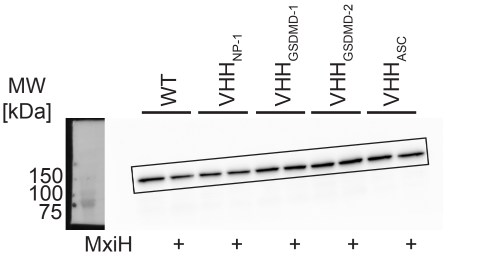

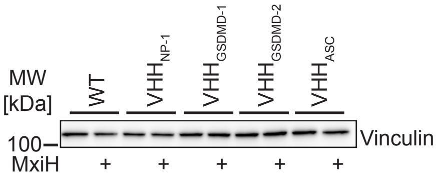

* These blots are all from the same lysates, just depicted in different figures in the manuscript. This is why the loading control occurs in multiple figures.

220811 Anti-caspase-3 (1:500)

Figure S5A

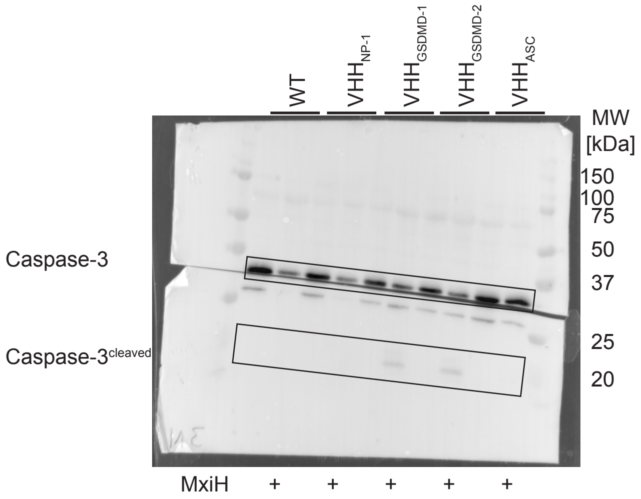

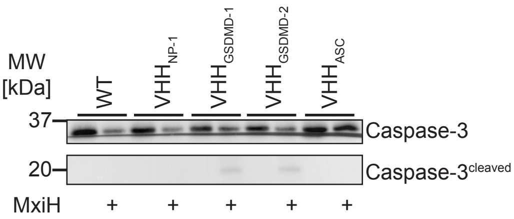

220929 Anti-caspase-7 (1:500)

Figure S5A

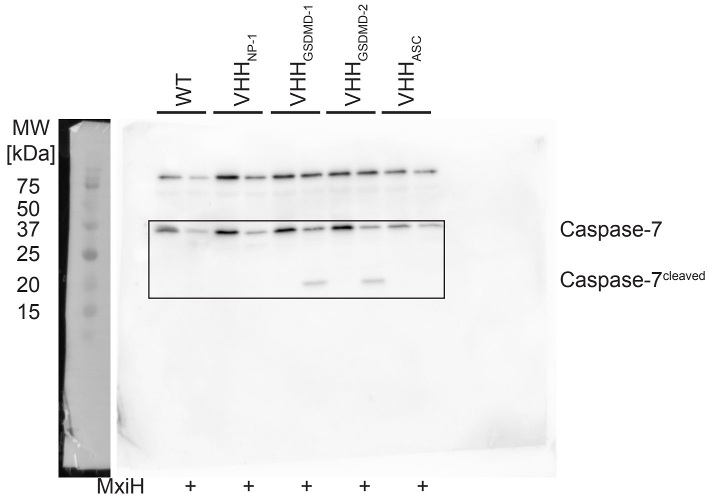

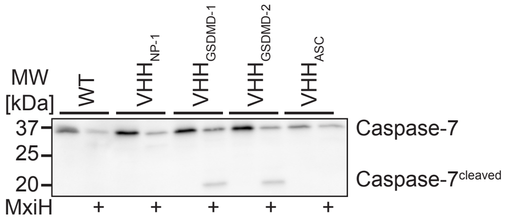

220630 Anti-GSDME (1:500)

Figure S5A

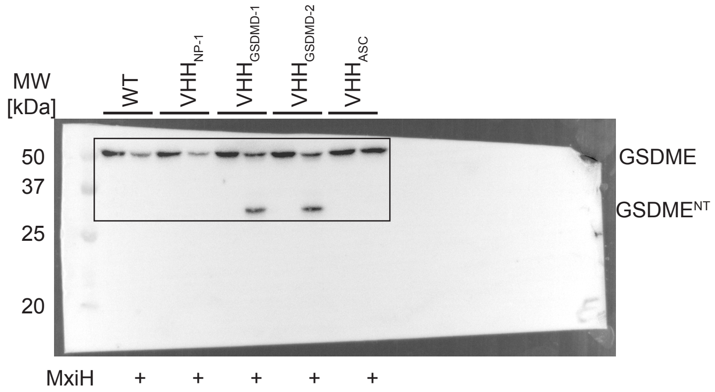

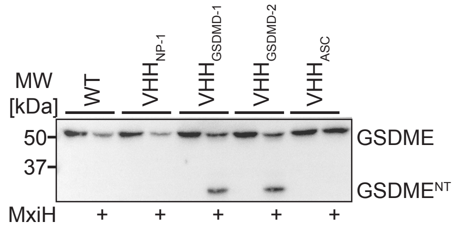

221721 Anti-PARP (1:500)

Figure S5A

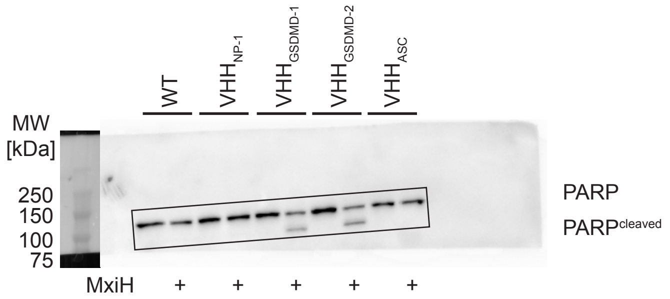

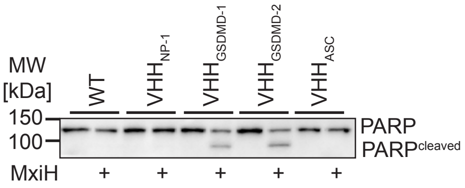

221006 Anti-vinculin (1:1000)

Figure S5A and S5D*

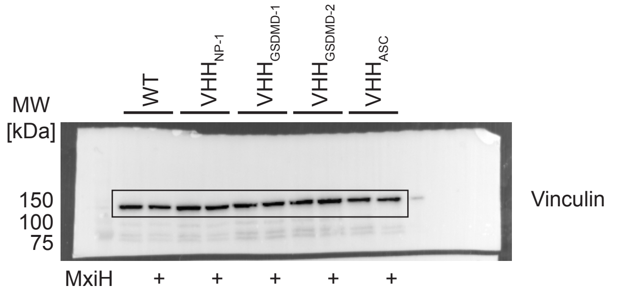

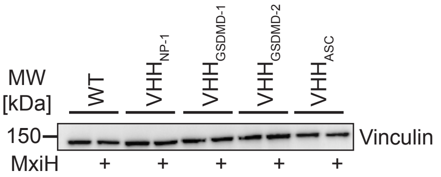

* These blots are all from the same lysates, just depicted in different figures in the manuscript. This is why the loading control occurs in multiple figures.

200721 Anti-GSDMD (1:500)

Figure S5B

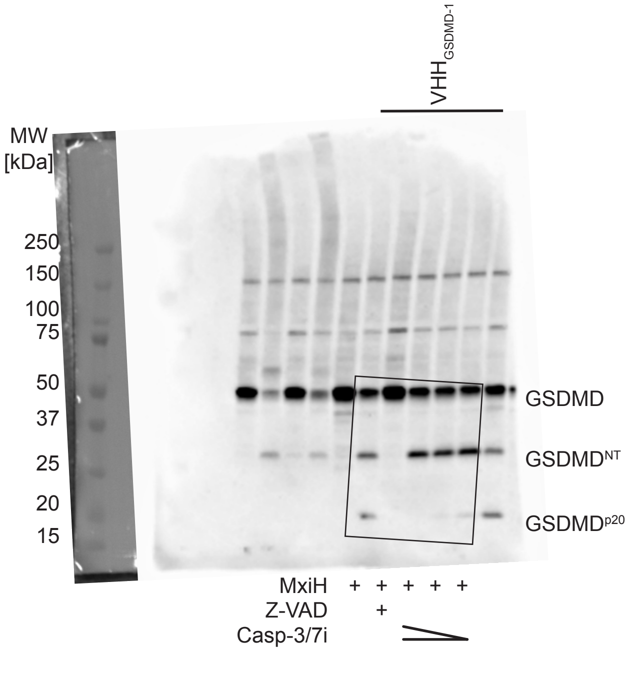

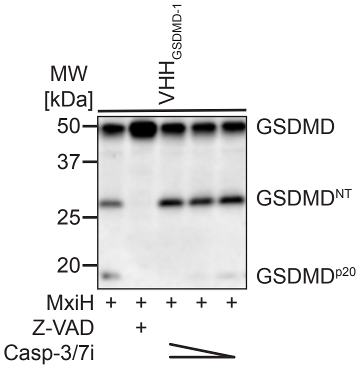

220811 Anti-caspase-8 (1:500)

Figure S5C

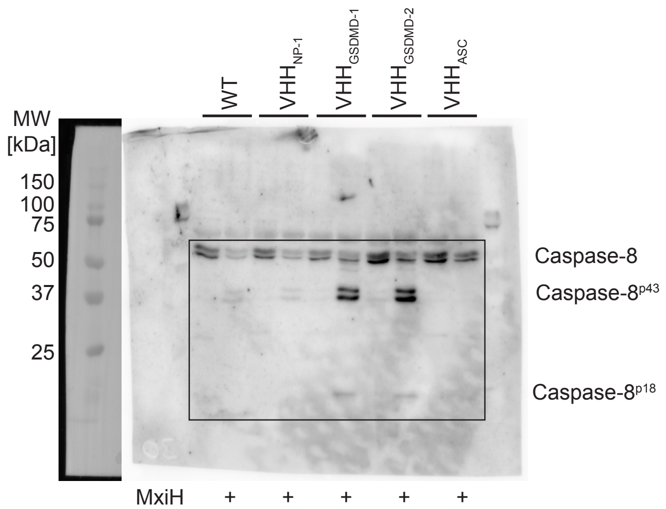

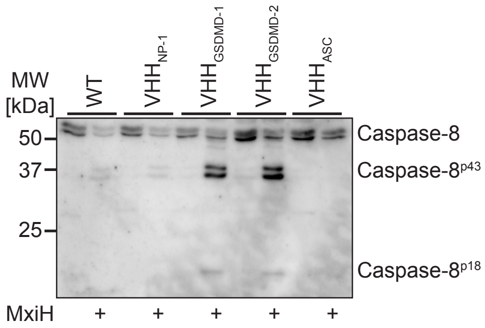

220915 Anti-caspase-9 (1:500)

Figure S5C

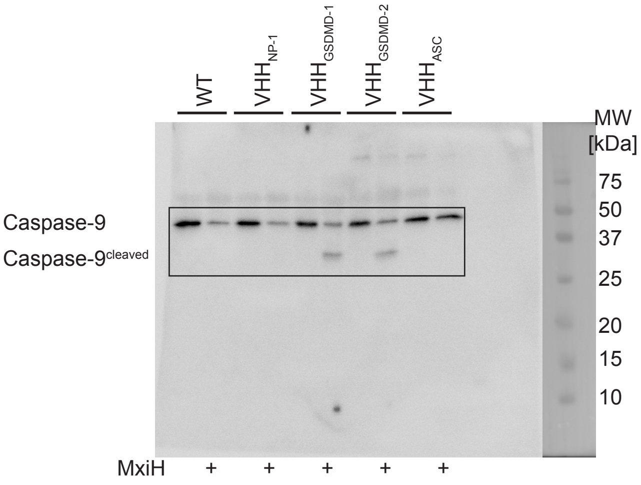

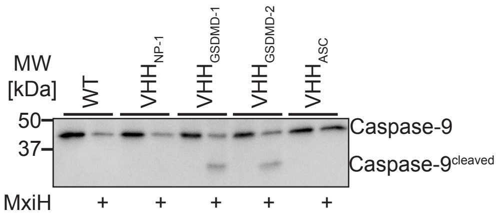

221006 Anti-BID (1:500)

Figure S5D

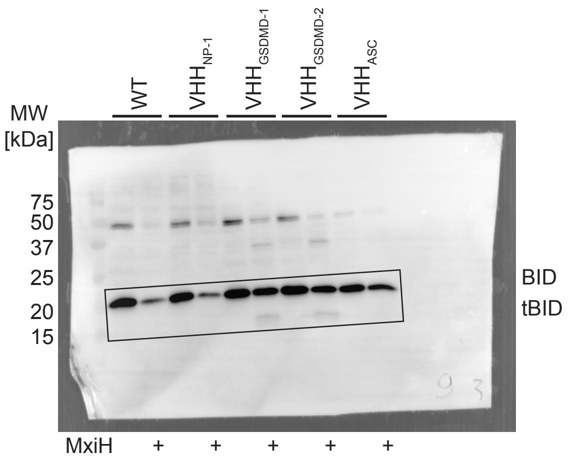

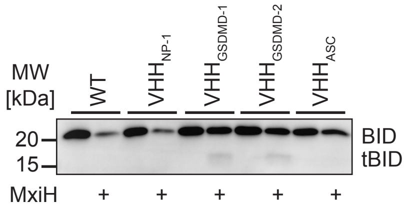

221114 Anti-BID (1:500)

Figure S5E

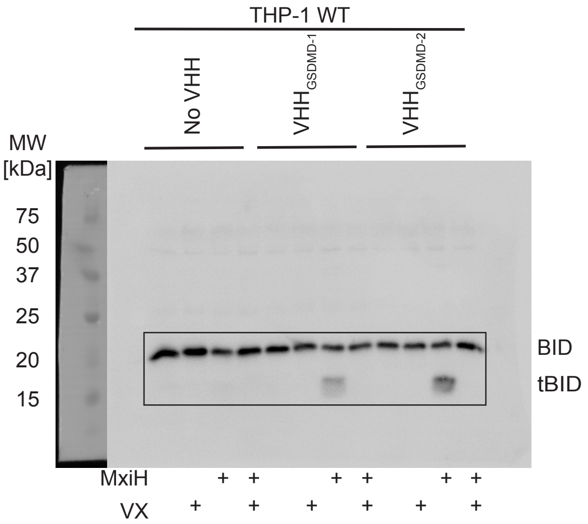

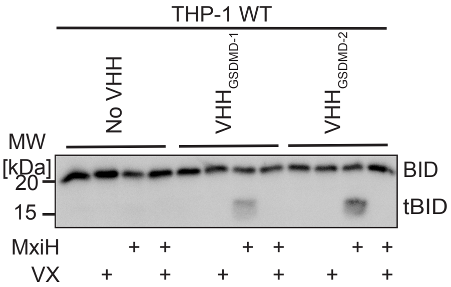

221107 Anti-caspase-8 (1:500)

Figure S5E

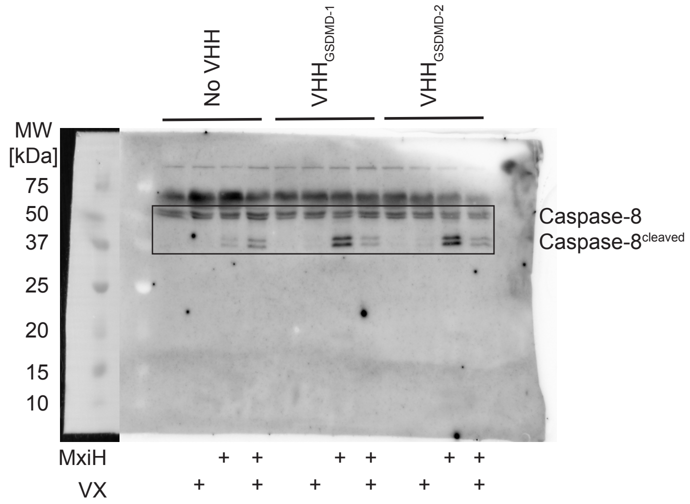

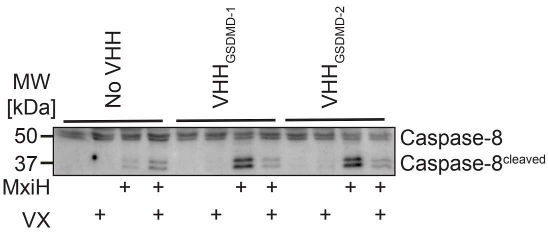

221107 Anti-caspase-9 (1:500)

Figure S5E

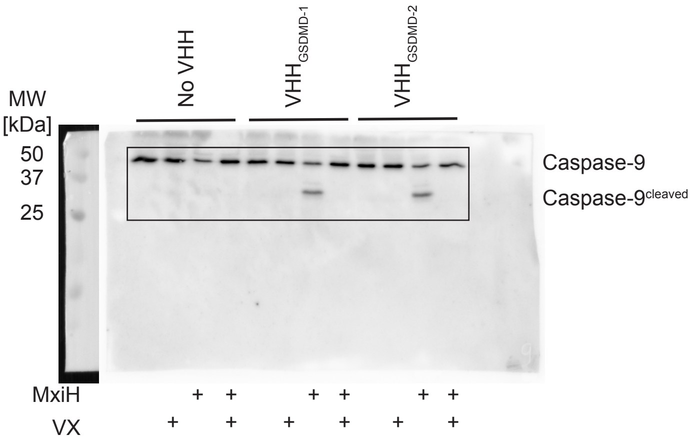

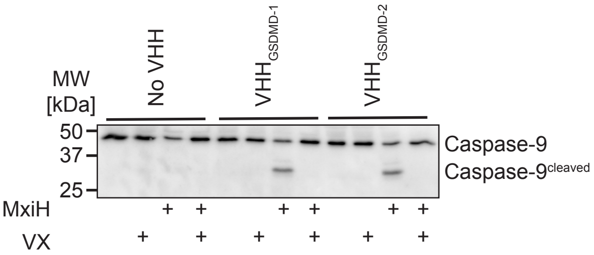

221103 Anti-vinculin (1:1000)

Figure S5E

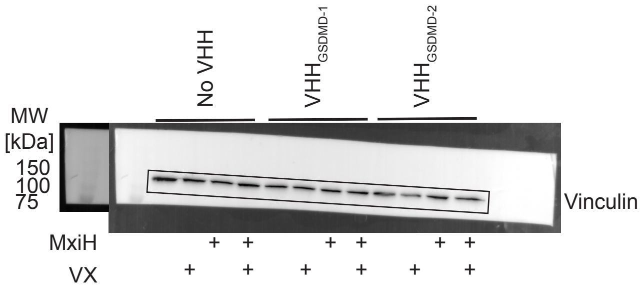

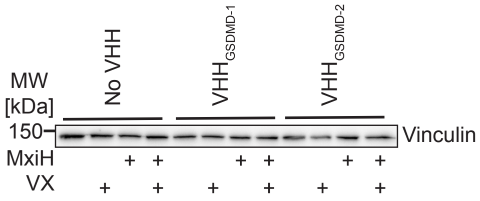

221114 Anti-BID (1:500)

Figure S5F

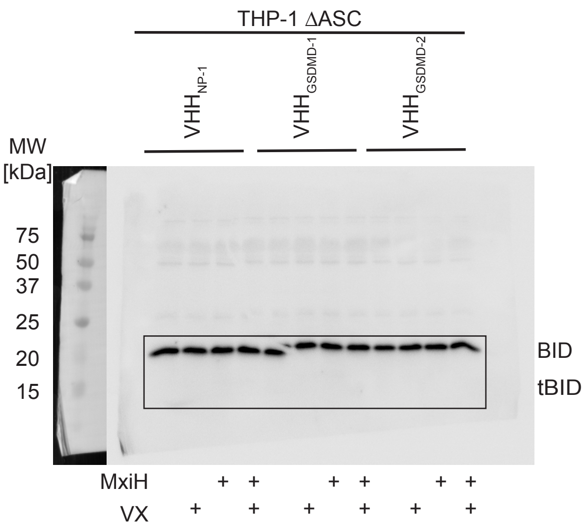

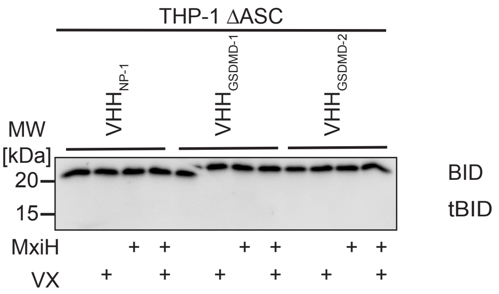

221107 Anti-caspase-8 (1:500)

Figure S5F

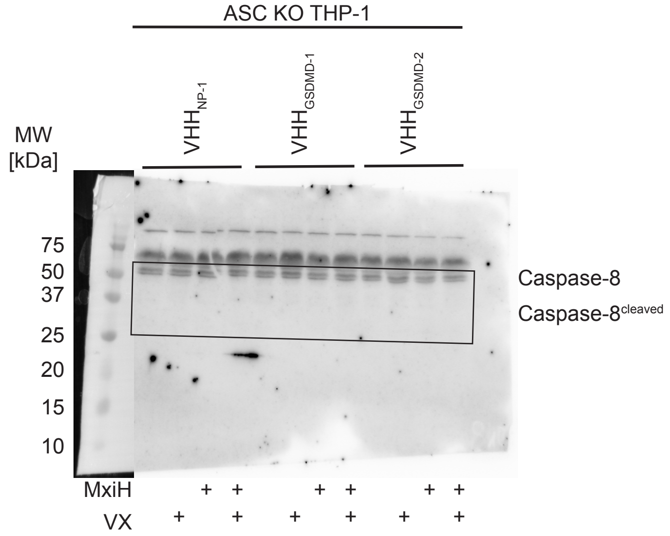

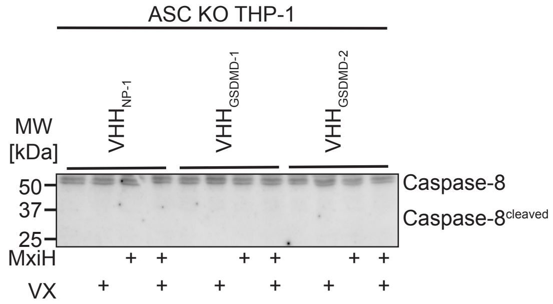

221107 Anti-caspase-9 (1:500)

Figure S5F

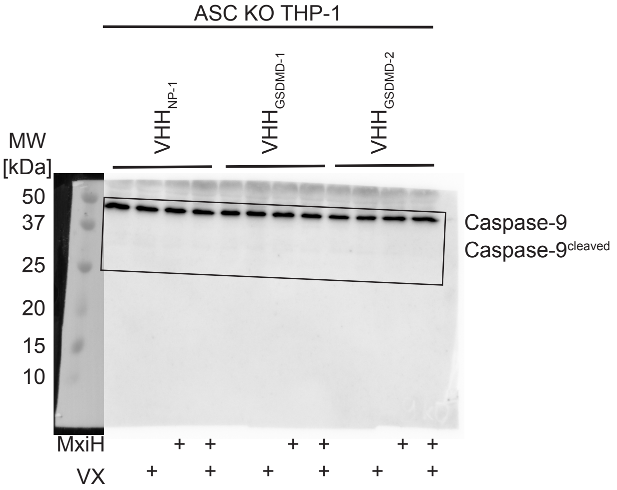

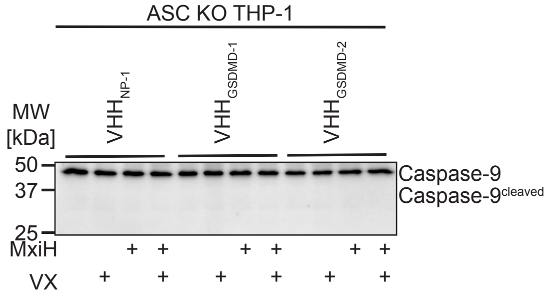

221103 Anti-vinculin (1:1000)

Figure S5F

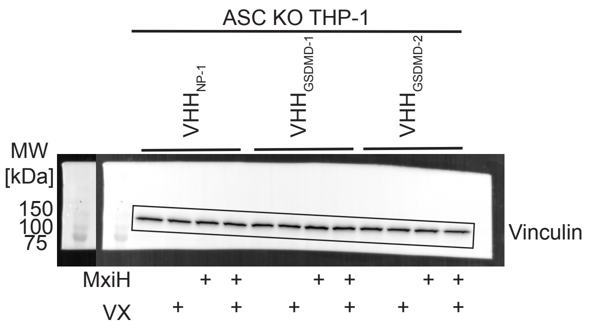

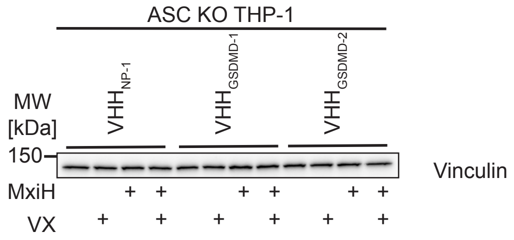

Supplement: Supplementary file 1 — Supplementary Information [file 41467_2024_52110_MOESM1_ESM.pdf]
