# Supplementary material for: Antagonistic nanobodies implicate mechanism of GSDMD pore formation and potential therapeutic application
Source: Nat Commun. 2024 Sep 26;15:8266. doi: 10.1038/s41467-024-52110-1 (PMC11427689; doi:10.1038/s41467-024-52110-1)
Supplement: Supplementary file 3 — Description of Additional Supplementary Files [file 41467_2024_52110_MOESM3_ESM.pdf]

Description of Additional Supplementary Files

Supplementary Movie 1. GSDMD^{NT}-mNG does not accumulate in the plasma membrane and is internalized in pyroptotic cells. PMA-differentiated THP-1 cells constitutively expressing GSDMD-mNG_ins (green) and VHH$_{NP-1}$-HA were stained with plasma membrane marker CMO (red), stimulated with MxiH as in Fig. 2C, and followed over time by live cell confocal microscopy (3 min intervals, time post treatment indicated). Selected time point of the same recording are displayed Fig. S4A. Scale bar, 5 µm.

Supplementary Movie 2. GSDMD^{NT}-mNG does not accumulate in the plasma membrane and is internalized in pyroptotic cells. PMA-differentiated THP-1 cells constitutively expressing GSDMD-mNG_ins (green) and VHH$_{NP-1}$-HA were treated and recorded as in Movie S1. Scale bar, 5 µm.

Supplementary Movie 3. Uptake of nanobodies through transient GSDMD pores precedes apoptosis. PMA-differentiated THP-1$^{C1C\text{-}tagBFP}$ were treated with MxiH in the presence of inhibitory VHH$_{GSDMD-1}$, low concentrations of VHH$_{ASC}$ AF647, and PI as described in Figure S10. Z stacks of cells with C1C-tagBFP specks were recorded over time by live cell confocal microscopy; the movie displays the focal planes indicated in Figure S10 (yellow arrows). Scale bar, 25 µm.

Supplementary Movie 4. Uptake of nanobodies through transient GSDMD pores precedes apoptosis. PMA-differentiated THP-1$^{C1C\text{-}tagBFP}$ were treated with MxiH in the presence of inhibitory VHH$_{GSDMD-1}$, low concentrations of VHH$_{ASC}$ AF647, and PI as described in Figure S11. Z stacks of cells with C1C-tagBFP specks were recorded over time by live cell confocal microscopy; the movie displays the focal planes indicated in Figure S11 (magenta arrows). Scale bar, 25 µm.

Supplementary Movie 5. Uptake of nanobodies through transient GSDMD pores precedes apoptosis. PMA-differentiated THP-1$^{C1C\text{-}tagBFP}$ were treated with MxiH in the presence of

inhibitory $VHH_{GSDMD-1}$, low concentrations of VHH_{ASC} AF647, and PI as described in Figure S11. Z stacks of cells with C1C-tagBFP specks were recorded over time by live cell confocal microscopy; the movie displays the focal planes indicated in Figure S11 (yellow arrows). Scale bar, 25 µm.

Supplementary Movie 6. Uptake of nanobodies through GSDMD during pyroptosis. PMA-differentiated $THP\text{-}1^{C1C\text{-}tagBFP}$ were treated with MxiH in the presence of control VHH_{NP-1}, low concentrations of VHH_{ASC} AF647, and PI as described in Figure S12. Z stacks of cells with C1C-tagBFP specks were recorded over time by live cell confocal microscopy; the movie displays the focal planes indicated in Figure S12 (yellow arrows). Scale bar, 25 µm.
